# Interplay between conformational selection and zymogen activation

**DOI:** 10.1038/s41598-018-21728-9

**Published:** 2018-03-06

**Authors:** Pradipta Chakraborty, Laura Acquasaliente, Leslie A. Pelc, Enrico Di Cera

**Affiliations:** Edward A. Doisy Department of Biochemistry and Molecular Biology, Saint Louis University School of Medicine, St. Louis, MO 63104 USA

## Abstract

Trypsin-like proteases are synthesized as zymogens and activated through a mechanism that folds the active site for efficient binding and catalysis. Ligand binding to the active site is therefore a valuable source of information on the changes that accompany zymogen activation. Using the physiologically relevant transition of the clotting zymogen prothrombin to the mature protease thrombin, we show that the mechanism of ligand recognition follows selection within a pre-existing ensemble of conformations with the active site accessible (E) or inaccessible (E*) to binding. Prothrombin exists mainly in the E* conformational ensemble and conversion to thrombin produces two dominant changes: a progressive shift toward the E conformational ensemble triggered by removal of the auxiliary domains upon cleavage at R271 and a drastic drop of the rate of ligand dissociation from the active site triggered by cleavage at R320. Together, these effects produce a significant (700-fold) increase in binding affinity. Limited proteolysis reveals how the E*-E equilibrium shifts during prothrombin activation and influences exposure of the sites of cleavage at R271 and R320. These new findings on the molecular underpinnings of prothrombin activation are relevant to other zymogens with modular assembly involved in blood coagulation, complement and fibrinolysis.

## Introduction

The trypsin family of proteases is widely distributed in nature and constitutes a significant component of a typical genome^1^. Members of this family participate in physiological processes such as digestion, blood coagulation, fibrinolysis, development, fertilization, apoptosis and immunity^2^. Nearly all of them are expressed as inactive zymogens that are irreversibly converted to the mature protease by proteolytic cleavage at an Arg residue in the activation domain^3^. The cleavage generates a new N-terminus that inserts into the protein and H-bonds to the side chain of the highly conserved D194 (chymotrypsinogen numbering). This interaction organizes the architecture of the entire active site, especially residue G193 in the oxyanion hole and the catalytic S195, and prepares the enzyme for substrate binding and catalysis^2,4,5^. The Huber-Bode mechanism of zymogen activation in the trypsin family described above is one of the most enduring paradigms in protease biology^3^. It rationalizes the onset of biological activity and is particularly useful in the interpretation of the initiation, progression and amplification of enzyme cascades^6,7^. The paradigm has also fostered the notion that activity associates with the protease (or protease-like states) and inactivity with the zymogen (or zymogen-like states)^4,5^. Yet, a number of zymogens spontaneously autoactivate^8–11^ or can be engineered to do so^12,13^ and a number of proteases are inactive unless bound to cofactors^14,15^. The zymogen may therefore access states featuring some level of activity, or protease-like, and the protease may assume conformations that are poorly active, or zymogen-like. These observations suggest that zymogen and protease are best described as points along a trajectory that connects active and inactive states within an ensemble of possible conformations. The exact active:inactive distribution along the trajectory describes the properties of zymogen and protease depending on biological context.

The plasticity of the trypsin fold within the ensemble is supported first and foremost by X-ray structural biology. The 215–217 segment defines the west wall of the active site and provides anchor points for substrate residues immediately upstream of the peptide bond to be cleaved by the protease^2,4,5^. A survey of high resolution (<3 Å) structures of proteases and zymogens currently deposited in the Protein Data Bank (PDB) documents multiple conformations for this segment, even in the same crystal^16–18^, that directly influence access to the active site^19,20^. Specifically, the Cα-Cα distance between the highly conserved residues G193 in the oxyanion hole and G216 in the 215–217 segment spans the range 7–13 Å and defines the aperture to the primary specificity pocket that requires >8 Å to ensure ligand binding to the active site. The structures in the PDB likely are snapshots of the active site region as it assumes conformations within the ensemble that either allow (E) or disallow (E*) binding as a necessary step for catalysis^19^. Structural evidence from the PDB is compelling but provides no proof that alternative conformations of the active site do exist in solution, nor it quantifies the E*-E distribution which remains a challenging task for both protease and zymogen. NMR measurements are ideally suited to address this task, but have so far been limited to the more rigid bound forms of the protease^21–24^ and only offered speculations about the free forms. Recent theoretical investigations of ligand binding mechanisms^25–27^ have renewed interest in rapid kinetics as a strategy to detect conformational transitions and whether they precede and/or follow the binding step. Rapid kinetic measurements of ligand binding to the active site of the protease^28^ support a mechanism through which optimal conformations are selected from a pre-existing ensemble, which is consistent with the PDB scenario. Whether the same mechanism applies to the zymogen precursor of the protease remains to be established.

In this study we extend our recent investigation of ligand binding to the active site of thrombin by studying its zymogen precursor prothrombin and elucidate the contribution of the E*-E pre-existing equilibrium as the zymogen transitions to the protease along its two possible pathways of activation^29^. We take advantage of the modular architecture of prothrombin^6^ to explore how its auxiliary Gla domain and kringles influence long range the properties of the active site in the protease domain. The results define a strategy of general applicability to other trypsin-like proteases and zymogens involved in blood coagulation, complement and fibrinolysis.

## Results

### Prothrombin activation

**Figure 1:**
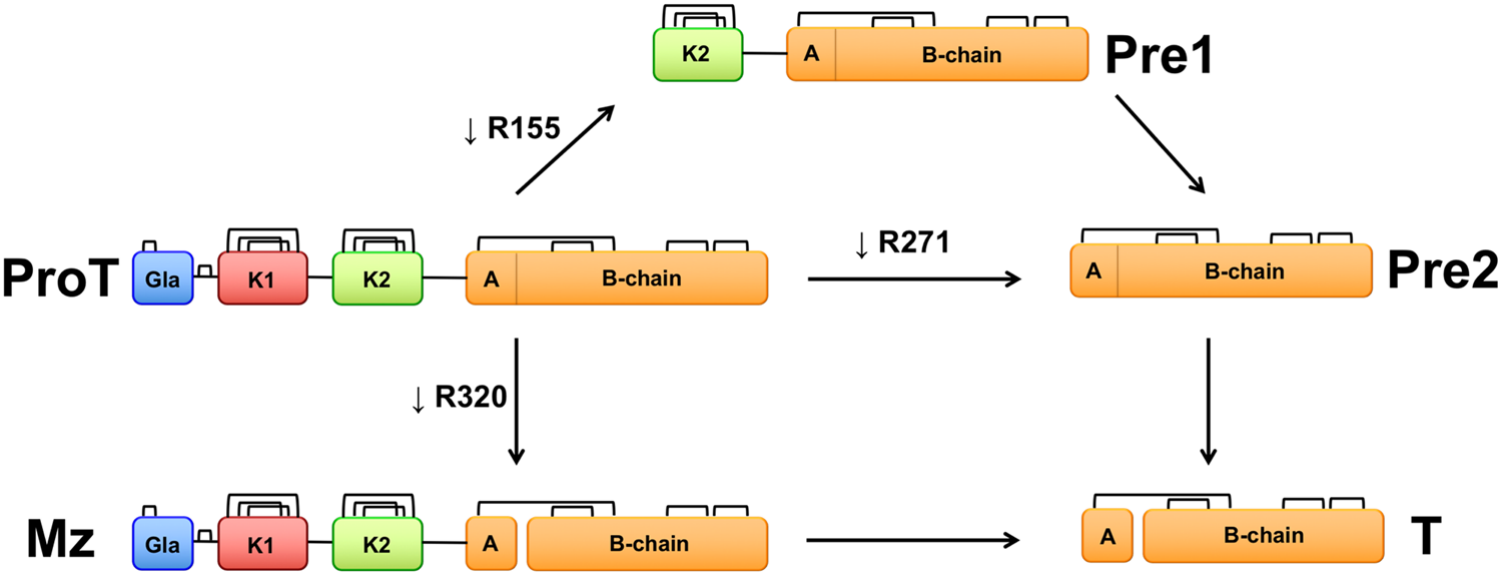
Cartoon representation of the various derivatives of prothrombin (ProT) produced upon activation. Prothrombin has a modular structure where the protease domain composed of the A and B chains (yellow) is linked in order to kringle-2 (K2, green), kringle-1 (K1, red) and the Gla domain (Gla, blue). The enzyme factor Xa preferentially cleaves prothrombin at R155 between the two kringles and generates the intermediate prethrombin-1 (Pre1) composed of kringle-2 and the protease domain. When factor Xa is assembled with cofactor Va in the prothrombinase complex, prothrombin is converted to thrombin (T) along two possible pathways. Cleavage at R271 sheds all auxiliary domains and generates the inactive intermediate prethrombin-2 (Pre2). Cleavage at R320 retains the modular structure of prothrombin and generates the active intermediate meizothrombin (MzT).

Prothrombin, or coagulation factor II, is composed of 579 residues and has a modular assembly (Fig. 1) that comprises the Gla-domain (A1-A46), kringle-1 (C65-C143), kringle-2 (C170-C248) and the protease domain (T272-E579)^30^. The Gla-domain confers the ability to bind to phospholipids on platelets, red blood cells and the endothelium, thereby localizing and enhancing activation of the zymogen for biological response. The kringles provide additional surface for interaction with the protein components of prothrombinase (enzyme factor Xa, cofactor Va) when prothrombin is converted to the active protease thrombin in the penultimate step of the coagulation cascade. The conversion involves two proteolytic cleavages at R271 and R320 that shed the Gla domain and two kringles and generate thrombin as a free protease domain. Cleavage at R271 sheds the Gla domain and both kringles to generate prethrombin-2, a direct zymogen precursor of thrombin from which it differs only in the intact activation domain. Cleavage at R320 generates the active intermediate meizothrombin that retains the modular organization of prothrombin but with the active site folded for catalysis as described by the Huber-Bode mechanism. The conversion of prethrombin-2 to thrombin involves only the protease domain. The conversion of prothrombin to meizothrombin involves the entire modular assembly. Analysis of these reactions by rapid kinetics of ligand binding to the active site affords a unique opportunity to explore the role of the pre-existing E*-E equilibrium during zymogen activation, with or without the presence of auxiliary domains.

### Rapid kinetics of ligand binding to thrombin and meizothrombin

**Figure 2:**
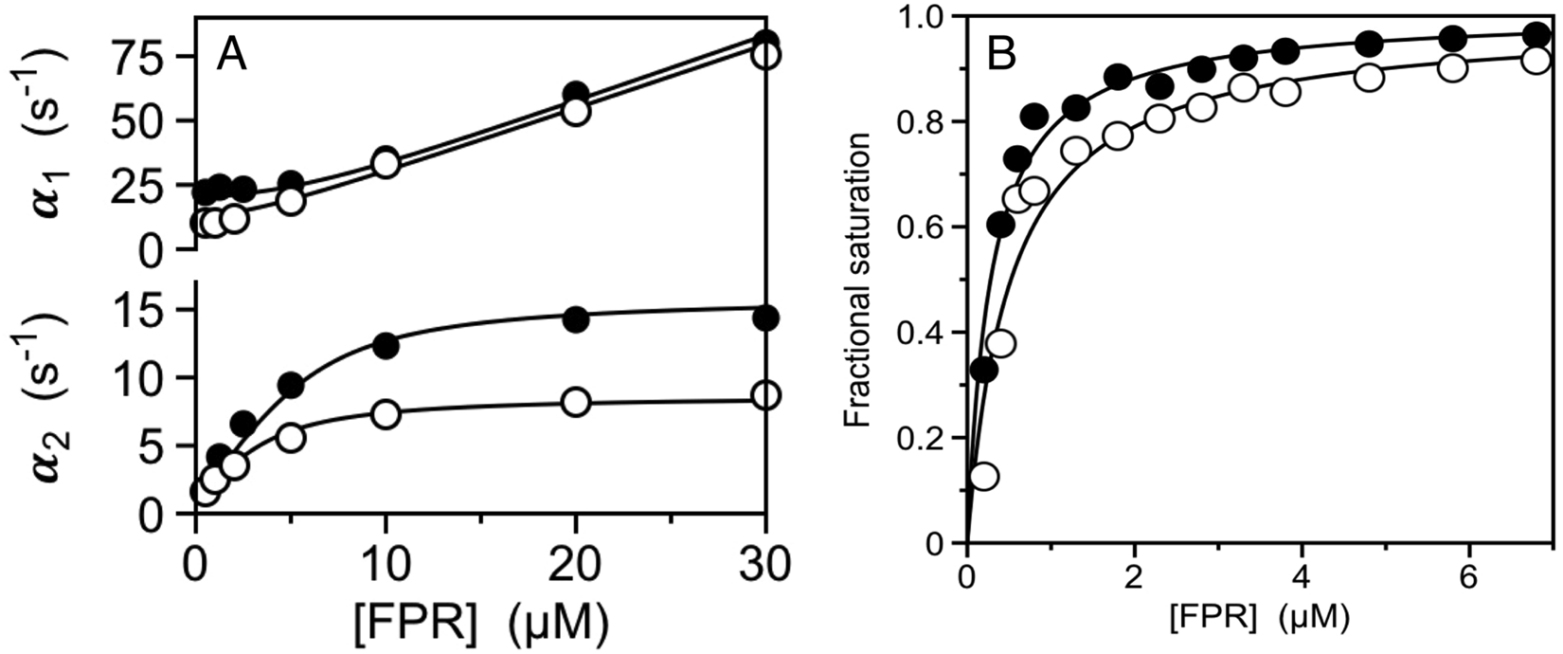
(**A**) Rapid kinetics of FPR binding to thrombin (closed circles) and meizothrombin (open circles) showing the two relaxations defining the mechanism of binding according to the reaction scheme in eqs 1 and 2. Continuous lines were drawn according to eq. 2 in the text with best-fit parameter values listed in Table 1. (**B**) Equilibrium titrations of FPR binding to thrombin (closed circles) and meizothrombin (open circles) consistent with a single-site binding interaction with best-fit values of *K*_*d*,*app*_ listed in Table 1. The kinetic and equilibrium properties of thrombin and meizothrombin are very similar and prove that the presence of auxiliary domains is inconsequential on ligand binding to the active site in the protease domain for the active forms. Experimental conditions are: 400 mM ChCl, 50 mM Tris, 0.1% PEG8000, pH 8.0, at 15 °C. Representative kinetic traces and residuals are shown in the Supplementary Information.

Figure 2A summarizes rapid kinetics data of FPR binding to the active site of thrombin and meizothrombin run under conditions of excess ligand. In both cases, the active site Ser was replaced by Ala to prevent hydrolysis of FPR and the interaction was followed as changes in intrinsic fluorescence of the protein. Binding of FPR to thrombin produces two relaxations that increase linearly (α_1_) and hyperbolically (α_2_) with the ligand concentration. In principle, this kinetic profile is consistent with the scheme in eq. 1 but also with an alternative mechanism of binding according to induced fit, as discussed in detail elsewhere^25,27^. However, measurements with excess macromolecule^28^ have already resolved this potential ambiguity in favor of the scheme in eq. 1 and analysis of the data according to eq. 2 produces rate constants *k*_12_ = 16 ± 1 s^−1^ and *k*_21_ = 3.8 ± 0.8 s^−1^, with a resulting E*:E ratio of 1:4 (Table 1). FPR binds to E with a 2^nd^-order rate constant *k*_*on*_ = 2.6 ± 0.3 μM^−1^s^−1^ and a dissociation rate constant *k*_*off*_ = 0.6 ± 0.1 s^−1^. The value of *K*_*d*,*app*_ = 290 ± 20 nM derived from these rate constants agrees with the value of *K*_*d*,*app*_ = 280 ± 20 nM derived independently from equilibrium measurements of intrinsic fluorescence titration (Fig. 2B). The data also are in close agreement with those reported recently under the same solution conditions^28^. FPR binding to meizothrombin (Fig. 2A) features a profile nearly identical to that of thrombin, with rate constants *k*_12_ = 8.8 ± 0.5 s^−1^, *k*_21_ = 3.4 ± 0.7 s^−1^ and a resulting E*:E ratio of 1:3 (Table 1). FPR binds to E with a 2^nd^-order rate constant *k*_*on*_ = 2.5 ± 0.3 μM^−1^s^−1^ and a dissociation rate constant *k*_*off*_ = 0.8 ± 0.1 s^−1^, giving *K*_*d*,*app*_ = 440 ± 30 nM in reasonable agreement with the value *K*_*d*,*app*_ = 510 ± 40 nM obtained from equilibrium measurements (Fig. 2B). The similarities between thrombin and meizothrombin indicate that, once R320 is cleaved, auxiliary domains become inconsequential on the properties of the active site as probed by FPR binding.

**Table 1:** Kinetic rate constants for FPR binding to prothrombin and its products.

	*k*_12_ (s^−1^)	*k*_21_ (s^−1^)	*k*_*on*_ (μM^−1^s^−1^)	*k*_*off*_ (s^−1^)	*K*_*d*,*app*_ (μM)^a^	*K*_*d*,*app*_ (μM)^b^	E*:E
Prothrombin	6.9 ± 0.3	≥38 ± 3	≥1.5 ± 0.1	≥45 ± 5	≥200 ± 20	190 ± 20	≥6:1
Prethrombin-1	42 ± 2	≥96 ± 8^c^	≥0.46 ± 0.05	42 ± 2	≥92 ± 8^c^	92 ± 8	≥2:1^c^
Prethrombin-2	170 ± 10	≥100 ± 10^c^	≥0.93 ± 0.08	27 ± 2	≥29 ± 2^c^	29 ± 2	≥1:2^c^
Meizothrombin	8.8 ± 0.5	3.4 ± 0.7	2.5 ± 0.3	0.8 ± 0.1	0.44 ± 0.03	0.51 ± 0.04	1:3
Thrombin	16 ± 1	3.8 ± 0.8	2.6 ± 0.3	0.6 ± 0.1	0.29 ± 0.02	0.28 ± 0.02	1:4

^a^Calculated from eq. 3. ^b^Determined by direct fluorescence titration. ^c^Calculated under the assumption that *k*_*on*_ = 1.5 μM^−1^ s^−1^. Experimental conditions are: 400 mM ChCl, 50 mM Tris, 0.1% PEG8000, pH 8.0, at 15 °C.

### Rapid kinetics of ligand binding to prothrombin

**Figure 3:**
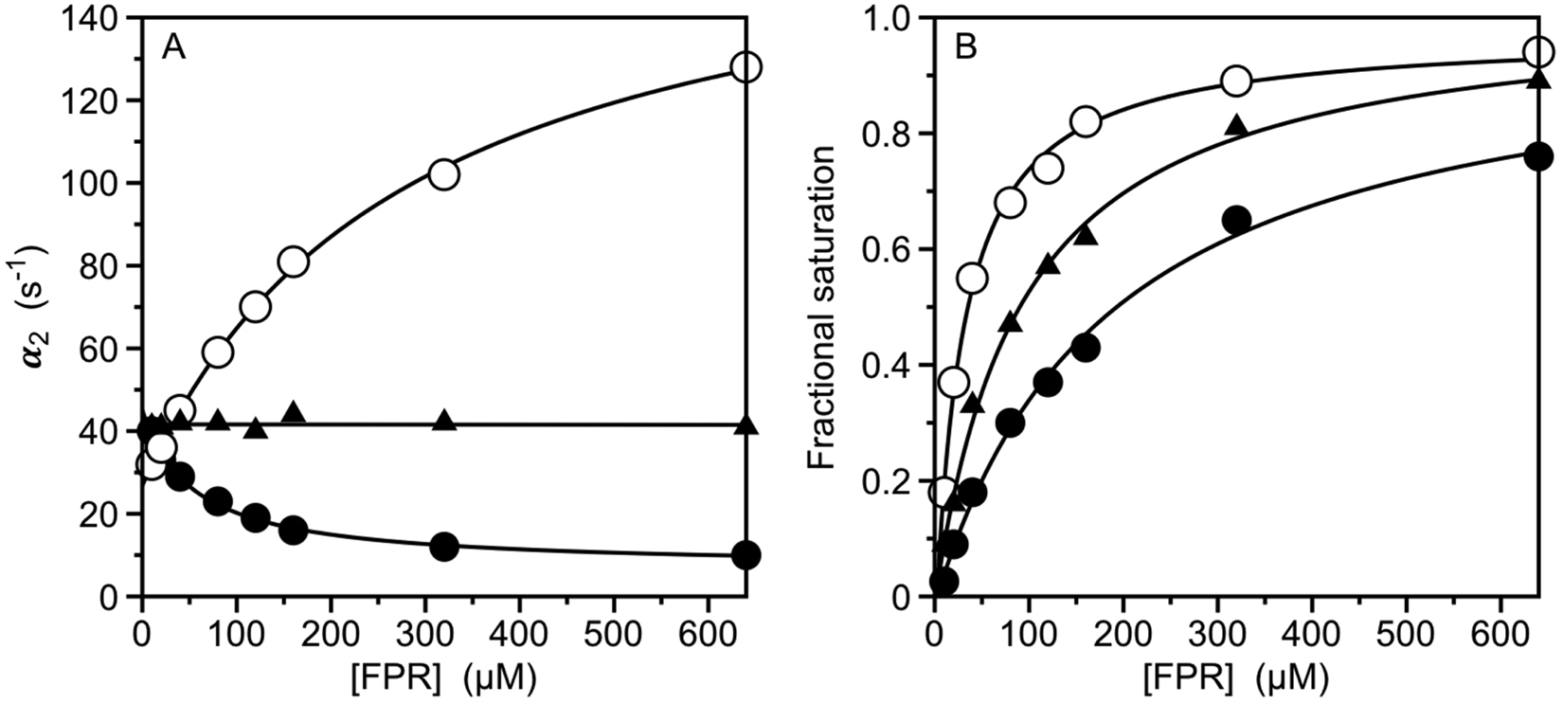
(**A**) Rapid kinetics of FPR binding to prothrombin (closed circles), prethrombin-1 (triangles) and prethrombin-2 (open circles) showing the slow relaxation *α*_2_ from the reaction scheme in eqs 1 and 2. Continuous lines were drawn according to eq. 2 in the text with best-fit parameter values listed in Table 1. (**B**) Equilibrium titrations of FPR binding to prothrombin (closed circles), prethrombin-1 (triangles) and prethrombin-2 (open circles) consistent with a single-site binding interaction with best-fit values of *K*_*d*,*app*_ listed in Table 1. The kinetic profiles of prothrombin, prethrombin-1 and prethrombin-2 differ markedly, especially in the value of *k*_12_. A progressive shift of the E*-E equilibrium toward the E conformational ensemble (Table 1) accounts for the increase in ligand binding affinity when auxiliary domains are removed in the zymogen from prothrombin to prethrombin-2. Experimental conditions are: 400 mM ChCl, 50 mM Tris, 0.1% PEG8000, pH 8.0, at 15 °C. Representative kinetic traces and residuals are shown in the Supplementary Information.

FPR binding to prothrombin produces a single relaxation decreasing hyperbolically with the ligand concentration (Fig. 3A) that proves unequivocally the validity of the reaction scheme in eq. 1 and rules out alternative mechanisms of binding such as induced fit^25^. The limiting values in the plot are α_2_(0) = 45 ± 5 s^−1^, defining the smaller between *k*_*off*_ or the sum *k*_12_ + *k*_21_, and ${\alpha }_{2}(\infty )={k}_{12}$ = 6.9 ± 0.3 s^−1^, with a resulting E*:E ratio ≥6:1 (Table 1). The significant difference in E*:E ratio between prothrombin and thrombin is due entirely to the value of *k*_21_being ≥10-fold higher in the zymogen. Access to the active site opens at the same rate (*k*_12_) in prothrombin and thrombin, but closes at a ≥10-fold faster rate (*k*_21_) in the zymogen. Ligand dissociation from the active site is ≥75-fold faster in prothrombin compared to thrombin. A lower estimate of the rate of ligand association is derived from analysis of FPR binding at equilibrium (Fig. 3B), that yields *K*_*d*,*app*_ = 190 ± 10 μM, and application of eq. 4. The resulting value of *k*_*on*_ ≥ 1.5 μM^−1^ s^−1^ shows a rate of ligand binding to the active site of prothrombin in the E conformational ensemble surprisingly similar to that of thrombin. Therefore, the 700-fold higher affinity of thrombin compared to prothrombin (Table 1) is explained by a slower closure of the active site in the E → E* transition and a slower dissociation rate in the mature enzyme, with the rates of opening of the active site in the E* → E transition and of ligand association being comparable between protease and zymogen. Overall, the conversion of prothrombin to thrombin shifts the E*-E equilibrium from ≥6-fold in favor of E* to 4-fold in favor of E and lowers the value of *k*_*off*_ for ligand dissociation from the active site.

### Rapid kinetics of ligand binding to prethrombin-1 and prethrombin-2

Information on the factors that control ligand binding to the active site of prothrombin comes from consideration of the role of auxiliary domains. Cleavage at R155 by factor Xa removes the Gla domain and kringle-1 and generates prethrombin-1 (Fig. 1). FPR binding to prethrombin-1 features a single relaxation independent of ligand concentration (Fig. 3A) that again proves unequivocally the validity of the reaction scheme in eq. 1^25^ and leads to the relationship ${\alpha }_{2}(0)={\alpha }_{2}(\infty )={k}_{off}={k}_{12}$ = 42 ± 2 s^−1^. The value compares well to ${\alpha }_{2}^{\,}(0)$ = 45 ± 5 s^−1^ measured for prothrombin and suggests that *k*_*off*_ does not change significantly upon removal of the Gla domain and kringle-1. On the other hand, the value of *k*_12_ measuring the rate of opening of the active site in the E* → E transition increases significantly in prethrombin-1 compared to prothrombin. The molecular origin of this effect deserves attention. Recent developments on the structure of prothrombin in solution have revealed an unanticipated intramolecular interaction of kringle-1 with the protease domain made possible by the intrinsic flexibility of the linker connecting the two kringles^31^. Removal of this interaction in prethrombin-1 may change the dynamics of the protease domain resulting in a faster time scale for the E* → E transition. Support to this scenario comes from removal of kringle-2 from prethrombin-1 by cleavage at R271 to generate prethrombin-2. In this case, FPR binding produces a single relaxation that increases hyperbolically with the ligand concentration (Fig. 3A). Again, measurements with excess macromolecule^28^ prove the validity of the scheme in eq. 1 and analysis of the data according to eq. 2 produces rate constants with ${\alpha }_{2}^{\,}(0)={k}_{off}$ = 27 ± 2 s^−1^ and ${\alpha }_{2}(\infty )={k}_{12}$ = 170 ± 10 s^−1^. The rate for the E* → E transition is even faster than that of prethrombin-1 suggesting that removal of kringle-2 further accelerates the dynamics of the protease domain. The drastic decrease in the value of *k*_12_ when prethrombin-2 converts to thrombin likely originates from rigidification of the active site region upon zymogen activation. The profiles of prethrombin-1 and prethrombin-2 (Fig. 3A) prevent unequivocal estimation of the E*:E ratio. However, the equilibrium binding curves for prethrombin-1 and prethrombin-2 yield values of *K*_*d*,*app*_ = 92 ± 8 μM and 29 ± 2 μM, respectively (Table 1, Fig. 3B) and application of eq. 5 for prethrombin-2 and eq. 6 for prethrombin-1 provides a lower estimate for *k*_*on*_ in the range observed for prothrombin and thrombin (Table 1). Hence, this rate constant changes little in the protease and zymogen, independent of auxiliary domains, and so does the value of *k*_*off*_ for all zymogen species. Estimates of the E*:E ratio for prethrombin-1 and prethrombin-2 require assumptions on the value of *k*_*on*_. Taking 1.5 μM^−1^ s^−1^ as reasonable lower limit for *k*_*on*_, the E*:E ratio can be estimated as ≥2:1 (*k*_21_ ≥ 96 ± 8 s^−1^) for prethrombin-1 and ≥1:2 (*k*_21_ ≥ 100 ± 10 s^−1^) for prethrombin-2 (Table 1), which positions the two derivatives along a trajectory where the E conformational ensemble is progressively stabilized during the transition of prothrombin to thrombin.

### Role of conformational selection in prothrombin activation

**Figure 4:**
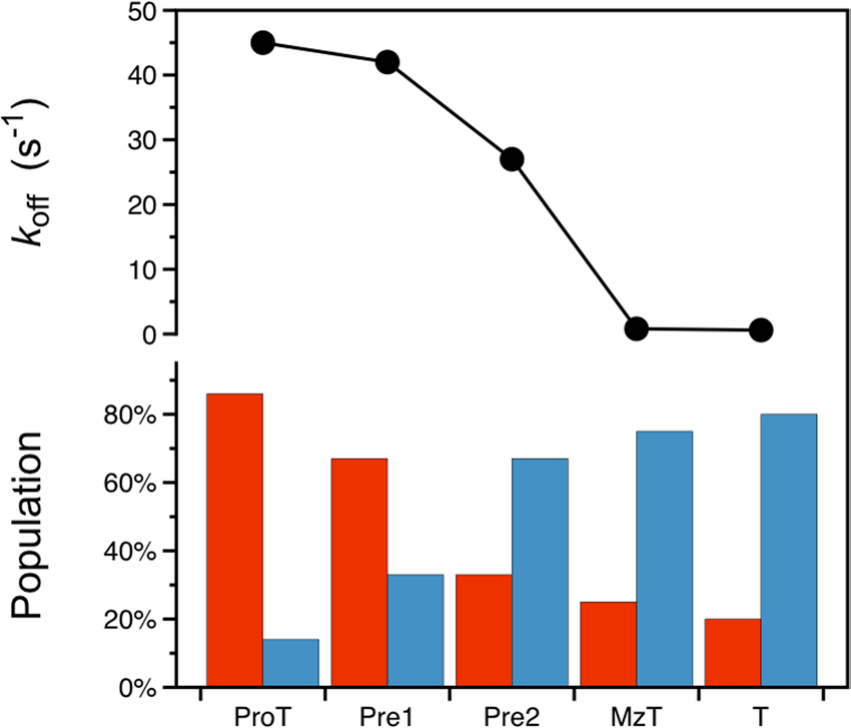
Components of the activation mechanism of prothrombin. (top) Value of the rate of ligand dissociation *k*_*off*_ for prothrombin (ProT) and its derivatives prethrombin-1 (Pre1), prethrombin-2 (Pre2), meizothrombin (MzT) and thrombin (T). The value drops drastically when cleavage at R320 triggers the Huber-Bode mechanism of activation and produces either meizothrombin or thrombin. (bottom) Distribution of E* (red bar) and E (blue bar) for prothrombin and its derivatives. The E* population gradually decreases as the auxiliary domains of prothrombin are removed in the zymogen and activation to the mature enzyme ensues. The values reported in the figure are derived from Table 1.

Activation of prothrombin to thrombin organizes the active site region and promotes catalysis. The changes envisioned by the Huber-Bode mechanism of activation^3^ can be given a functional interpretation in terms of a shift of the E*-E equilibrium in favor of E and a drastic drop in the rate of ligand dissociation (Fig. 4). Together, the effects produce a significant (700-fold) increase in binding affinity for the ligand prior to catalysis. In the case of prothrombin, the bulk of these changes take place after cleavage at R320 in the activation domain and generation of meizothrombin. The alternative cleavage at R271 sheds all auxiliary domains and shifts the E*-E distribution in favor of E but produces no significant drop in the value of *k*_*off*_. Hence, cleavage in the activation domain at R320 along the meizothrombin pathway according to the Huber-Bode mechanism selectively triggers higher binding affinity by slowing the rate of ligand dissociation. Redistribution of the E*-E equilibrium in favor of E is triggered by either cleavage at R271 (prethrombin-2 pathway) or R320 (meizothrombin pathway). Consequently, the auxiliary domains of prothrombin control the E*-E equilibrium in the zymogen but play little role upon activation. The value of ligand dissociation drops drastically upon activation and is influenced little by the presence of auxiliary domain.

### Limited proteolysis studies

**Figure 5:**
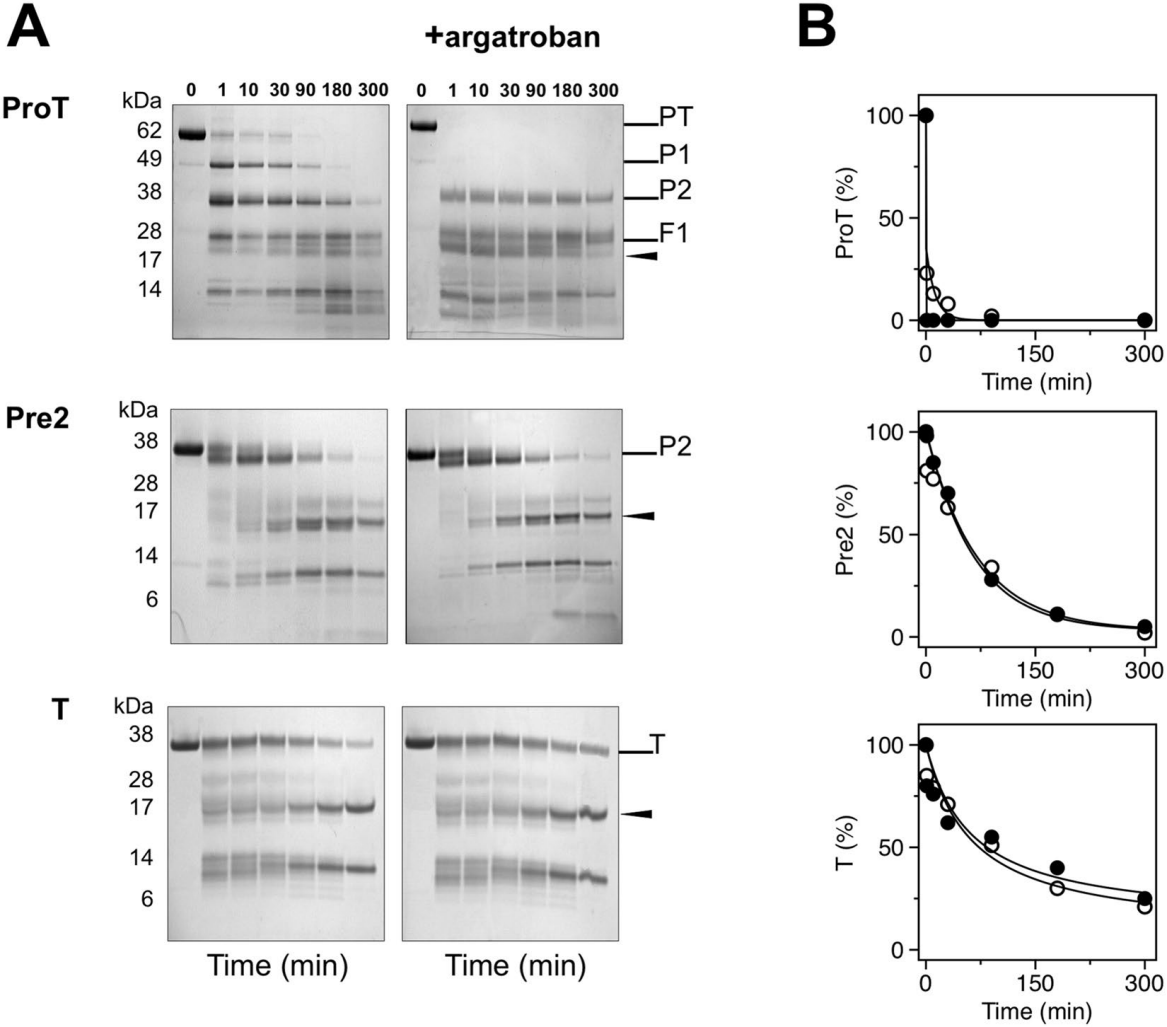
(**A**) Time course of the proteolysis of prothrombin (ProT), prethrombin-2 (Pre2) and thrombin (T) by subtilisin, under non-reducing conditions, in the absence and presence of the active site inhibitor argatroban. Subtilisin cleaves A470 in the autolysis loop (arrow) only when prethrombin-2 is generated. Argatroban affects proteolysis of prothrombin and prevents accumulation of prethrombin-1 (P1) but not fragment 1 (F1, Gla domain plus kringle-1). (**B**) Exponential time course of the proteolytic digestion of prothrombin, prethrombin-2 and thrombin by subtilisin obtained by densitometric analysis of the gels in the absence (open circles) and presence (filled circles) of argatroban. Binding of argatroban drastically (>10-fold) enhances the rate of prothrombin digestion but has no effect on prethrombin-2 and thrombin, in line with the shift in the E*-E distribution observed by rapid kinetics (Fig. 4). Gels were not cropped and originals are shown in the Supplementary Information.

**Figure 6:**
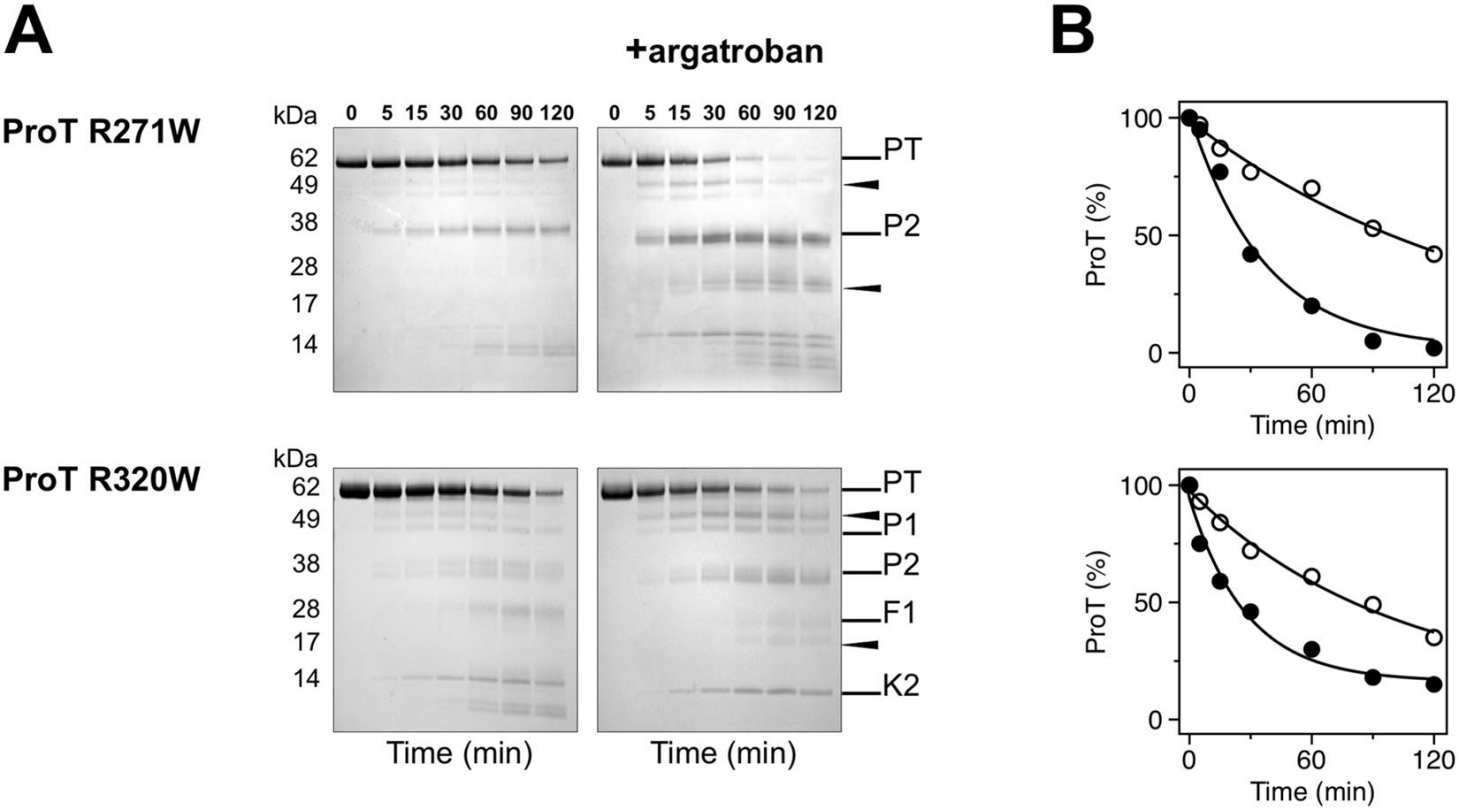
(**A**) Time course of the proteolysis of the prothrombin mutants R271W and R320W (0.1 mg/ml) with chymotrypsin, under non-reducing conditions, in the absence and presence of the active site inhibitor argatroban. In the wild-type, cleavage by chymotrypsin at W468 (arrow) only occurs in the argatroban-bound form but not in the free form^31^. The same result is observed for the R271W and R320W mutants. (**B**) Exponential time course of the reactions in the absence (open circles) and presence (filled circles) of argatroban obtained by densitometric analysis of the gels. Binding of argatroban enhances the rate of chymotrypsin hydrolysis >3-fold (${k}_{\exp }$ = 0.027 ± 0.004 min^−1^ vs 0.008 ± 0.001 min^−1^ for R271W, ${k}_{\exp }$ = 0.035 ± 0.007 min^−1^ vs 0.010 ± 0.003 min^−1^ for R320W). Gels were not cropped and originals are shown in the Supplementary Information.

Independent support to the role of conformational selection revealed by rapid kinetics comes from limited proteolysis experiments. Interest in this aspect of zymogen function comes from the recent observation that prothrombin is proteolytic attacked by subtilisin at several residues to produce prethrombin-1, prethrombin-2 and eventually a derivative of prethrombin-2 cleaved at A470 in the flexible autolysis loop that is activated independent of the Huber-Bode mechanism^32^. The rate of proteolytic digestion of prothrombin by subtilisin is significantly enhanced in the presence of the active site inhibitor argatroban (Fig. 5), which binds to the zymogen^31^ and switches the E*-E distribution in favor of the E conformational ensemble. The effect indicates that the conformation of prothrombin probed by subtilisin changes significantly upon binding to the active site and that the free form of the zymogen is dominated by the E* conformational ensemble, as detected by rapid kinetics. Interestingly, subtilisin cleavage does not accumulate prethrombin-1 in the presence of argatroban. This implies that the linker connecting the two kringles (Lnk2), which houses the cleavage site at R155 for generation of prethrombin-1^29^, has different exposure to solvent in the E* and E conformations. The observation confirms the role of the flexible Lnk2 in making the overall structure of prothrombin very dynamic with kringle-1 and the protease domain coming into transient intramolecular interaction, as unraveled recently by X-ray crystallography and single molecule spectroscopy^31,33–35^. The effect of argatroban on subtilisin digestion of prethrombin-2 is much less pronounced than that of prothrombin and practically absent in thrombin (Fig. 5). Hence, the effect of active site ligation on the proteolytic attack by subtilisin progressively decreases when prothrombin transitions to thrombin, in agreement with the increasing population of E relative to E* detected by rapid kinetics (Fig. 4). The E*-E equilibrium also affects accessibility of the two sites of cleavage of prothrombin by prothrombinase, i.e., R271 and R320 (Fig. 1). Because argatroban inhibits prothrombinase, the two sites were mutated individually to Trp to enable selective cleavage by chymotrypsin, that has no appreciable activity toward wild-type prothrombin^31^ or binding affinity for argatroban. The prothrombin mutants R271W and R320W are cleaved by chymotrypsin selectively at the Trp residues introduced in the protein and with rates that increase 3-fold in the presence of argatroban (Fig. 6). This proves that the solvent exposure of R271 and R320 increases upon the E* → E transition of prothrombin.

## Discussion

Observational evidence from the PDB points to the trypsin fold as an ensemble of conformations with different accessibility of the active site^19,20^. Structural determinants of this effect include the 215–217 segment^18–20,36^, the flexible autolysis loop^18,33–35,37^ and the 99-loop^38^ among other factors reported recently^39^. Transitions within the ensemble likely occur over a wide range of time scales and rapid kinetics are limited to detection of events unfolding within ms (Figs 2 and 3). Evidence of a pre-existing equilibrium between conformations that prevent (E*) or allow (E) binding to the active site is unequivocal within this time resolution^28^. Rapid rearrangements of the bound complex may not be excluded but, if present, they occur over a much faster time scale. Conformational selection in terms of the E*-E equilibrium can be quantified by rapid kinetics in both protease and zymogen and represents an important functional component of the celebrated Huber-Bode mechanism of activation^3^. In the specific case of the conversion of prothrombin into thrombin in the penultimate step of the coagulation cascade, activation produces a shift of the E*-E equilibrium from E* to E and a drastic drop in the rate of ligand dissociation from the active site that altogether account for a 700-fold increase in binding affinity. Independent measurements with limited proteolysis reveal a pattern of digestion in prothrombin, prethrombin-2 and thrombin that tracks along the shift from E* to E during activation. The E*-E equilibrium also affects exposure of the three sites of cleavage of prothrombin at R155, R271 and R320, thereby revealing important new information about the mechanism of prothrombin activation.

A significant implication of ligand recognition according to the scheme in eq. 1 is that accessible and inaccessible states of the active site co-exist in solution for thrombin and its inactive precursor prothrombin. Previous rapid kinetics and single molecule spectroscopy measurements^28,31,40–42^ also support this conclusion and so does the entire PDB database of trypsin-like proteases and zymogens^19,20^. Hence, the transition between “zymogen-like” E* conformations to “protease-like” E conformations pre-exists in solution. This fundamental property of the trypsin fold has obviously escaped so-called “alternative” explanations of thrombin function claiming that free thrombin exists exclusively in a zymogen-like conformation^43^ and that ligand binding transitions thrombin from zymogen-like to proteinase-like states^44^. We note that experimental support for these “alternative” narratives is rather shaky. Discussions of the zymogen-like nature of free thrombin have used structures of the E* form^44–46^ and conveniently ignored all structures of free thrombin^36,47^ and other trypsin-like proteases^19,20^ in the E form. NMR studies of progressively rigidified bound thrombin have offered only speculations about the zymogen-like nature of free thrombin and reported no assignments for this form^22^. Finally, proponents of ligand induced shuttling of thrombin and its precursors along a “continuum of zymogen-like and proteinase-like states”^44^ have yet to provide convincing experimental evidence of the validity of this mechanism by rapid kinetics or equivalent means.

Conformational selection in the trypsin fold has physiological relevance. Poorly active proteases, like coagulation factor VIIa^14^ or complement factor D^15^, are likely stabilized in the E* conformational ensemble and shift to E upon binding of cofactors. Poorly active variants of clotting factor Xa bypass the intrinsic pathway of coagulation and ameliorate hemophilia conditions^48^. The variants likely exist in the E* form until binding of cofactor Va restores activity by switching the conformational ensemble to E. These proteases should be studied by rapid kinetics to establish if their mechanism of action obeys the reaction scheme in eq. 1. Mutations in the 215–217 segment of thrombin produce variants that are poorly active toward all substrates except the thrombomodulin-protein C complex and offer interesting opportunities as protein anticoagulants^49–53^. Crystal structures of some of these variants document the collapsed E* form^46,54^ and rapid kinetics measurements confirm stabilization of the E* conformational ensemble in solution^27^. Transition to E is triggered selectively upon binding of thrombomodulin and protein C. In the zymogen, the presence of even small fractions of the E conformational ensemble may enable the minuscule catalytic activity necessary to trigger autoactivation either spontaneously^8–11,55–57^ or induced by external factors^12,13,58–60^.

## Methods

### Reagents

Prothrombin wild-type and mutants R271W and R320W, prethrombin-1, prethrombin-2, meizothrombin and thrombin were expressed as previously described^18,35,47^. The variants used for stopped-flow measurements were expressed with the catalytic Ser replaced by Ala to prevent hydrolysis of the chromogenic substrate H-D-Phe-Pro-Arg-p-nitroanilide (FPR) used as active site ligand. FPR was purchased from Bachem.

### Limited proteolysis

Proteins (0.1 mg/ml) were reacted with sequencing grade chymotrypsin (Promega) at 1:50 (w/w) ratio or with subtilisin from *Bacillus licheniformis* (Sigma) at 1:1000 (w/w) ratio, in 10 mM Tris, 145 mM NaCl, 5 mM CaCl_2_, pH 7.4 at 37 °C, in the absence or presence of the active site inhibitor argatroban (500 μM). Aliquots of proteolysis mixtures were quenched whit NuPAGE LDS buffer and analyzed by non-reducing SDS-PAGE (4–12% acrylamide). None of the gels were cropped and originals are available in the Supplementary Information. The relative intensity of intact protein gel bands, after Coomassie staining, was estimated by densitometric analysis and fit to single exponential decay over time.

### Stopped-flow experiments

Rapid kinetic experiments were conducted on an Applied Photophysics SX20 stopped-flow spectrometer under conditions of excess ligand. The dead time of the mixing cell for this instrument is 0.5–1 ms. Final concentrations of 50–75 nM of thrombin and meizothrombin, or 200–400 nM of prothrombin, prethrombin-1 and prethrombin-2 were used in a buffer containing 400 mM ChCl, 50 mM Tris, 0.1% PEG8000, pH 8.0, at 15 °C. The solution containing the protein was mixed 1:1 with 60 µL solutions of FPR in the same buffer. FPR is the cleavable analog of the irreversible active site inhibitor H-D-Phe-Pro-Arg-CH_2_Cl, for which detailed structural information exists on its interaction with the active site of thrombin^47,61^. Rapid kinetics of FPR binding were studied using an excitation of 295 nm and a cutoff filter at 320 nm. Baselines were measured by mixing the protein into buffer in the absence of ligand. Each kinetic trace for a given ligand concentration was taken as the average of a minimum of six determinations. Traces were fit to single or double exponentials based on the analysis of residuals using software supplied by Applied Photophysics.

### Mechanism of binding

A detailed discussion of ligand binding mechanisms established by rapid kinetics is given elsewhere^25–27^. and is briefly summarized below for the sake of completeness. The relevant kinetic scheme for ligand binding to the active site of a trypsin-like protease or zymogen is

1 $${{\rm{E}}}^{\ast }\begin{array}{c}{k}_{12}\\ \mathop{\leftarrow }\limits^{\longrightarrow }\\ {k}_{21}\end{array}{\rm{E}}\begin{array}{c}{k}_{on}[{\rm{L}}]\\ \mathop{\leftarrow }\limits^{\longrightarrow }\\ {k}_{off}\end{array}{\rm{E}}:{\rm{L}}$$

E* and E depict the partitioning of the ensemble of pre-existing conformations between those with the active site accessible (E) or inaccessible (E*) to ligand binding. The ligand, L, selectively binds to E with a second order rate of association *k*_*on*_. Under conditions where L is in large excess over the macromolecule, the reaction scheme in eq. 1 gives two independent rates of relaxation to equilibrium according to the expression

2 $$2{\alpha }_{1,2}={k}_{12}+{k}_{21}+{k}_{on}[{\rm{L}}]+{k}_{off}\pm \sqrt{{({k}_{on}[{\rm{L}}]+{k}_{off}-{k}_{12}-{k}_{21})}^{2}+4{k}_{21}{k}_{on}[{\rm{L}}]}$$

The fast relaxation, *α*_1_, always increases and eventually grows linearly with [L]. Depending on the sign of the expression ${k}_{off}^{\,}-{k}_{12}$, the value of the slow relaxation *α*_2_ hyperbolically decreases (${k}_{off}^{\,} > {k}_{12}$) or increases (${k}_{off}^{\,} < {k}_{12}$) with [L], and remains constant when ${k}_{off}^{\,}={k}_{12}$^25–27^. The fast relaxation that eventually grows linearly with [L] reflects the binding event. The slow relaxation monitors the conformational transition associated with binding.

### Estimating the E*:E ratio

Resolution of all four independent rate constants in the reaction scheme in eq. 1 requires measurements of both relaxations. In this case, as seen for thrombin and meizothrombin (Fig. 2A) the values of *k*_12_ and *k*_21_ estimate the E*:E ratio ${k}_{21}/{k}_{12}$. When only the slow relaxation *α*_2_ is available because the fast relaxation is spectroscopically silent or too fast to detect by stopped-slow, as seen for prothrombin, prethrombin-1 and prethrombin-2 (Fig. 3A), only a few of the four independent rate constants in eq. 2 can be resolved unequivocally. When *α*_2_ decreases hyperbolically with [L], the lower limit in the plot, ${\alpha }_{2}(0)$, measures the value of *k*_*off*_ or the sum ${k}_{12}+{k}_{21}$, whichever is smaller. The upper limit ${\alpha }_{2}(\infty )$ measures the rate for the E* → E transition *k*_12_. In this case, as seen for prothrombin (Fig. 3A), the E*:E ratio can be estimated from the values of the two limits in the plot as $\ge \frac{{\alpha }_{2}^{\,}(0)-{\alpha }_{2}^{\,}(\infty )}{{\alpha }_{2}^{\,}(\infty )}$, and the value of *k*_*off*_ is ≥*α*_2_ (0) (Table 1). When *α*_2_ increases hyperbolically with [L], as seen for prethrombin-2 (Fig. 3A), the lower limit in the plot, ${\alpha }_{2}(0)$, measures the rate of ligand dissociation *k*_*off*_ and the upper limit, ${\alpha }_{2}(\infty )$, again measures *k*_12_. When *α*_2_ is independent of [L], as seen for prethrombin-1 (Fig. 3A), then ${\alpha }_{2}(0)={\alpha }_{2}(\infty )={k}_{off}={k}_{12}$. If only *α*_2_ is accessible to experimental measurements, estimation of the E*:E ratio requires additional information from the apparent equilibrium dissociation constant

3 $$\,{K}_{d,app}=\frac{{k}_{off}}{{k}_{on}}(1+\frac{{k}_{21}}{{k}_{12}})=\,{K}_{d}(1+\frac{{k}_{21}}{{k}_{12}})$$

defined in terms of the intrinsic equilibrium dissociation constant, *K*_*d*_, and the E*:E ratio $\frac{{k}_{21}^{\,}}{{k}_{12}}.\,$Direct and independent determination of *K*_*d*,*app*_ can be obtained by fluorescence titrations under the same experimental conditions used for stopped-flow measurements. The definition of *K*_*d*,*app*_ in eq. 3 leads to three useful expressions, i.e.,

4 $${k}_{on}\ge \frac{{\alpha }_{2}^{2}(0)}{{\alpha }_{2}(\infty )\,{K}_{d,app}}$$

when *α*_2_ decreases hyperbolically with [L] as in the case of prothrombin (Fig. 3A),

5 $${k}_{on}=\frac{{\alpha }_{2}(0)[{\alpha }_{2}(\infty )+{k}_{21}]}{{\alpha }_{2}(\infty )\,{K}_{d,app}}=\frac{{\alpha }_{2}(0)}{\,{K}_{d,app}}+\frac{{\alpha }_{2}(0)}{{\alpha }_{2}(\infty )\,{K}_{d,app}}{k}_{21}$$

when *α*_2_ increases hyperbolically with [L] as in the case of prethrombin-2 (Fig. 3A), and

6 $$\,{k}_{on}=\frac{{\alpha }_{2}(0)+{k}_{21}}{\,{K}_{d,app}}=\frac{{\alpha }_{2}(0)}{\,{K}_{d,app}}+\frac{1}{\,{K}_{d,app}}{k}_{21}$$

when *α*_2_ is independent of [L] as in the case of prethrombin-1 (Fig. 3A). The expression in eq. 4 yields a direct estimate of the lower limit of *k*_*on*_ from which the value of *k*_21_ and the E*:E ratio can be arrived at with the help of eq. 3 (Table 1). The expressions in eqs 5 and 6, on the other hand, require assumptions to be made on the value of *k*_*on*_ to arrive at the value of *k*_21_. In both cases, a lower limit for *k*_*on*_ is estimated as ${k}_{on}\ge \frac{{\alpha }_{2}(0)}{\,{K}_{{\rm{d}},{\rm{app}}}}$.

### Data and Materials Availability

Recombinant reagents and data presented in this study are available from the corresponding author upon reasonable request.

## Electronic supplementary material

Supplementary Information
