# Supplementary material for: Interplay between conformational selection and zymogen activation
Source: Sci Rep. 2018 Mar 6;8:4080. doi: 10.1038/s41598-018-21728-9 (PMC5840343; doi:10.1038/s41598-018-21728-9)

Supplementary Materials

Interplay between conformational selection and zymogen activation

Pradipta Chakraborty, Laura Acquasaliente, Leslie A. Pelc and Enrico Di Cera

Edward A. Doisy Department of Biochemistry and Molecular Biology, Saint Louis University School of Medicine, St. Louis, MO 63104 USA

Kinetic traces and residuals for thrombin, meizothrombin, prothrombin, prethrombin-1 and prethrombin-2 whose values of k_{obs} are shown in Figures 2 and 3.

Raw gels shown in Figures 5 and 6.

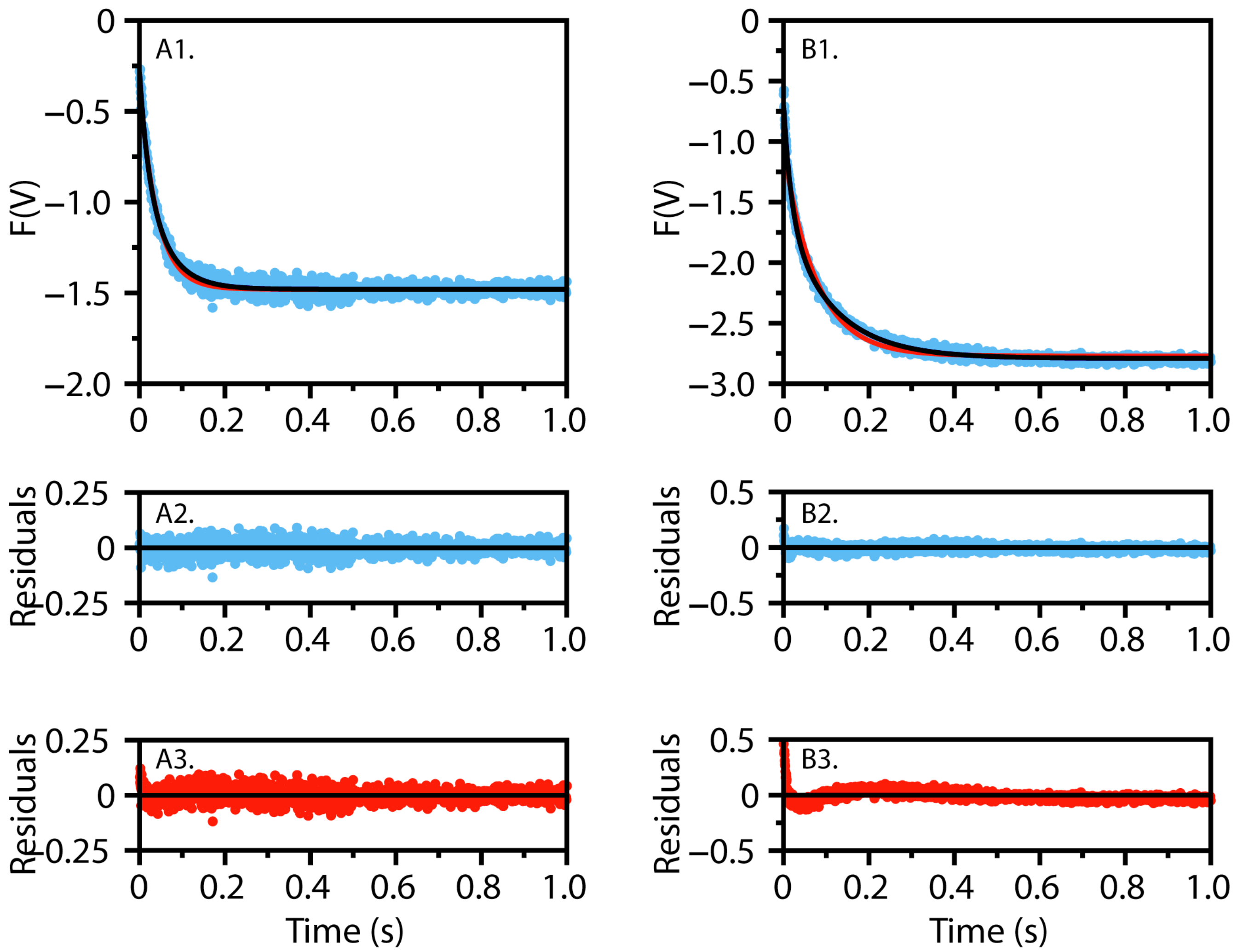

FPR (15 μM) binding to thrombin (A1) or meizothrombin (B1) fit to single (red) or double (black) exponential relaxations with resulting residuals (A2, B2=double-exponential fit, A3, B3=single exponential fit).

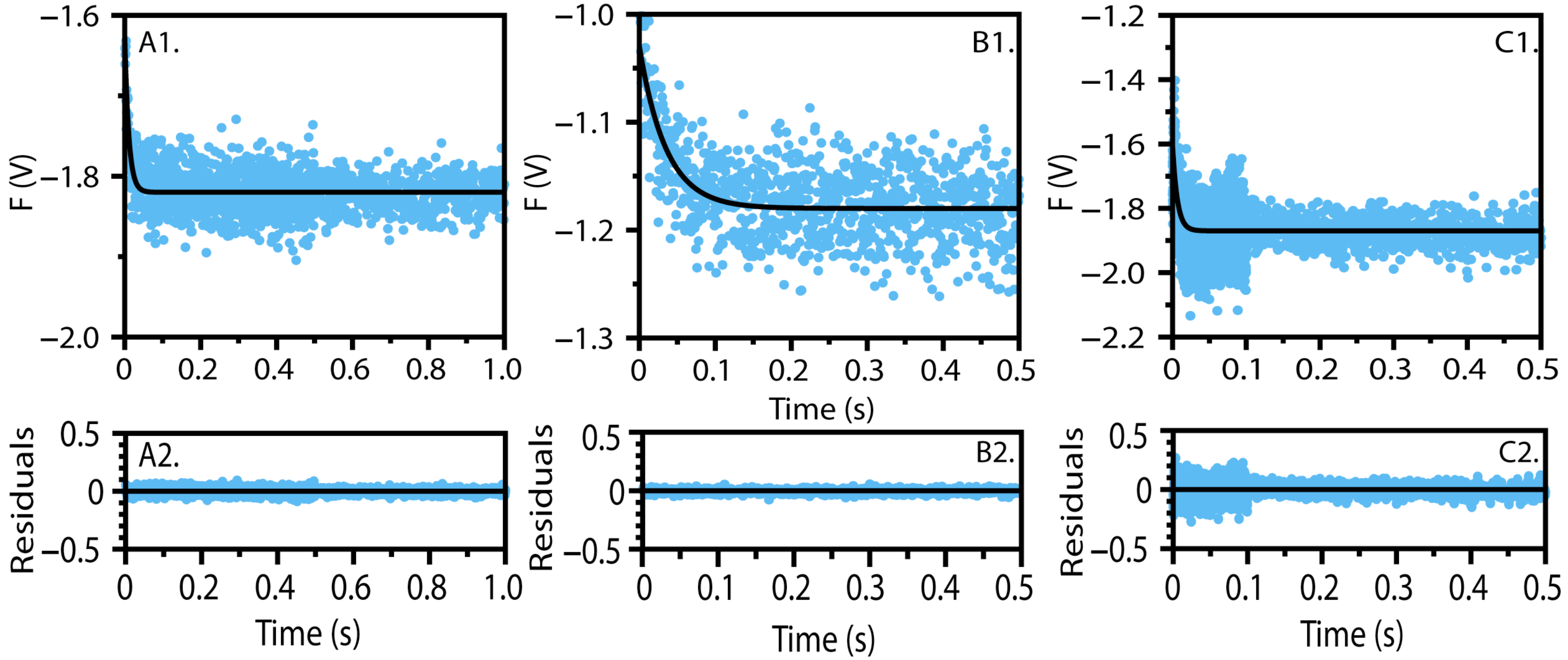

FPR (320 μM) binding to prothrombin (A1), prethrombin-1 (B1) or prethrombin-2 (C1) fit to a single exponential relaxation with resulting residuals (A2, B2, C2).

Figure 5_raw gels

+argatroban

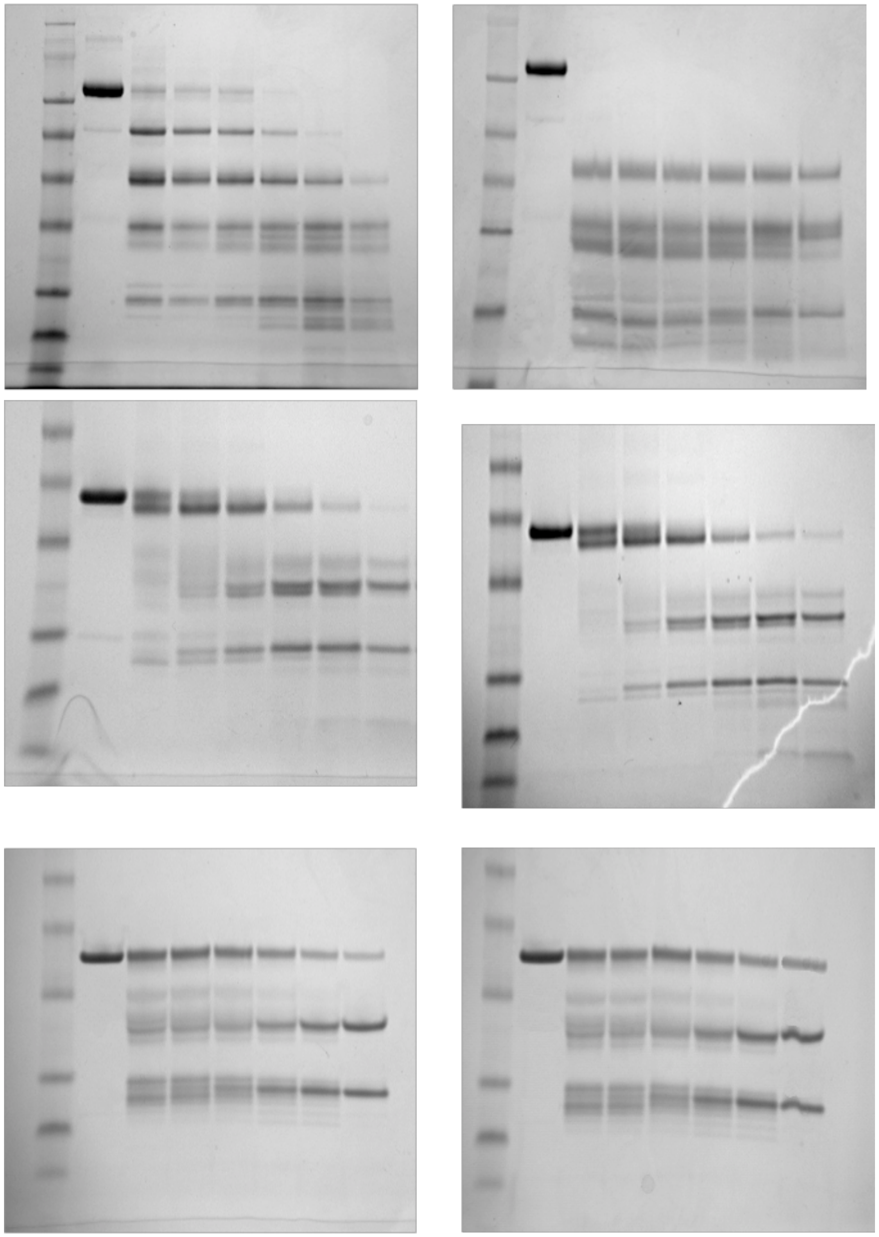

Figure 6_raw gels

+argatroban

ProT R271W

ProT R320W

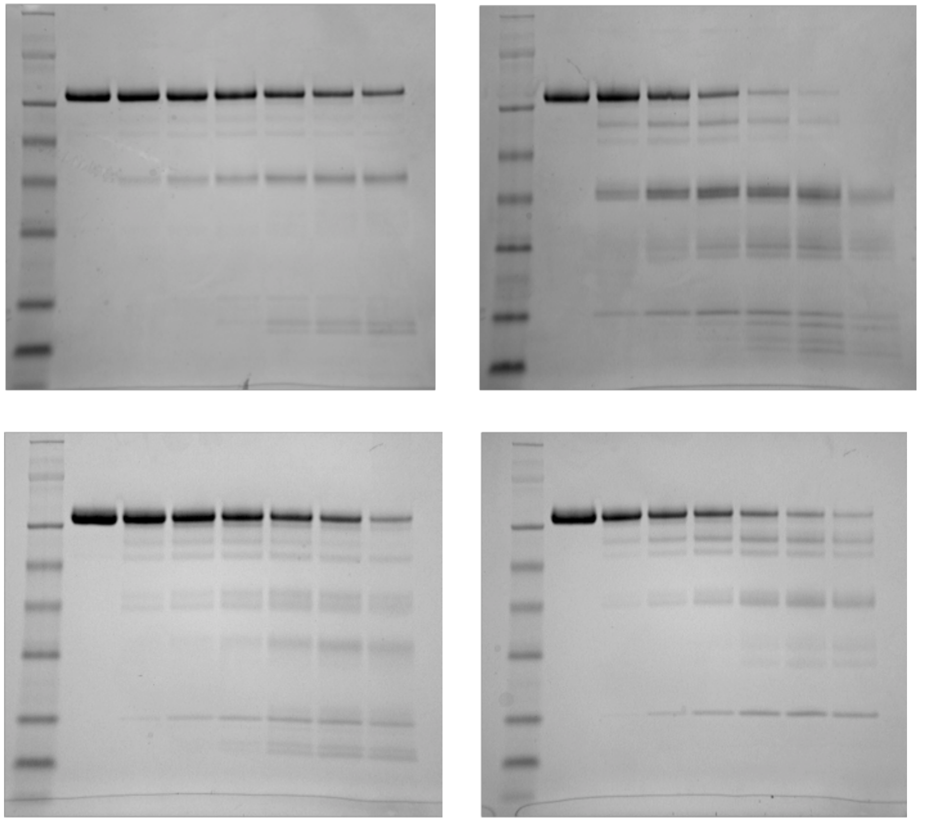

Supplement: Supplementary file 1 — Supplementary Information [file 41598_2018_21728_MOESM1_ESM.pdf]
